# A Novel Insight into the Role of Obesity-Related Adipokines in Ovarian Cancer—State-of-the-Art Review and Future Perspectives

**DOI:** 10.3390/ijms26051857

**Published:** 2025-02-21

**Authors:** Klaudia Kołakowska, Joanna Kiśluk, Jacek Nikliński

**Affiliations:** Department of Clinical Molecular Biology, Medical University of Białystok, 15-269 Białystok, Poland; kkolakowska.research@gmail.com (K.K.);

**Keywords:** adipokines, adipose tissue, apelin, chemerin, ovarian cancer, resistin, visfatin, cancer metastasis, metastasis biomarkers, cancer microenvironment

## Abstract

Ovarian cancer (OC) is one of the most fatal gynecological neoplasms. Meta-analyses have shown that the relationship between body mass index (BMI) and ovarian cancer incidence was detected in some types of ovarian cancer. Chronic inflammation and excessive accumulation of free fatty acids are key adipose tissue-derived factors initiating cancer development. Cancer cells transform adipose-derived stem cells into cancer-associated adipocytes, which produce adipokines and interleukins. It was revealed that adipokines exert a pleiotropic role in ovarian cancer pathogenesis. Chemerin presents both pro-cancer and anti-cancer action in ovarian cancer development. Chemerin induces angiogenesis and increases programmed death ligand-1 (PD-L1) expression, leading to enhanced proliferation and migration of OC cells. Apelin impacts cancer cell migration and acts as a mitogenic factor. Moreover, apelin exerts influence on lipid uptake into cancer cells and accelerates fatty acid oxidation, which provides energy for cancer cells. Visfatin induces matrix metallopeptidase 2 (MMP2) expression involved in extracellular matrix degradation and suppresses claudin 3 and 4 expression. Visfatin also induces a shift to anaerobic glucose metabolism and influences poly-ADP ribose polymerase (PARP). Resistin induces MMP2 and vascular endothelial growth factor (VEGF) expression and contributes to cisplatin-resistance development. A substantial body of evidence indicates that antagonists of adipokines mitigate OC progression, and adipokines are gaining gradual recognition as a potential therapeutic aim in ovarian cancer targeted therapy.

## 1. Introduction

**Figure 1:**
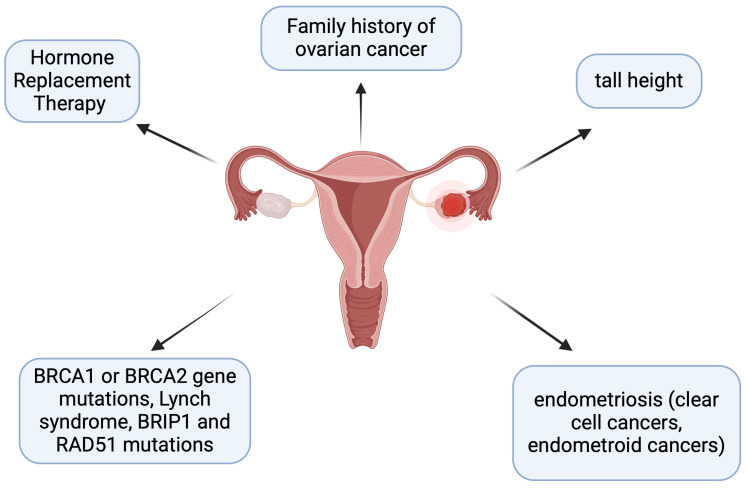
Ovarian cancer (OC) risk factors [3]. Created in BioRender. Kołakowski, A. (2025). https://BioRender.com/x72f623 (accessed on 9 July 2024).

Ovarian cancer (OC) is the eighth most common cancer in women and is the fifth most common cause of cancer-related fatality in women [1]. The vast majority of ovarian cancer cases are diagnosed at the advanced stage, with 5-year overall survival below 45% [2]. The number of ovarian cancer cases diagnosed each year is increasing in low-incidence countries but decreasing in high-incidence countries [3]. The OC risk factors are mentioned in Figure 1 [3].

The rapidly increasing worldwide prevalence of obesity constitutes a serious threat to the development of obesity-related malignancies and puts obesity in the limelight as a significant cancer risk factor. It is estimated that about 11.9% of cancer cases in men and 13.1% of cancer cases in women are correlated with the incidence of obesity [4]. A substantial correlation between higher body mass index (BMI) and a risk of cancer was demonstrated in endometrial, colorectal, gastric, liver, bladder, and prostate cancer [5]. However, similar studies pertinent to ovarian cancer yield inconsistent results. It is still not established whether there is a link between the risk of ovarian cancer and the obesity epidemic. Meta-analyses indicated that there is only a slight correlation between OC risk and higher BMI [6]. Moreover, the relationship between obesity and the risk of OC was only proved in some types of OC, i.e., borderline tumors, invasive endometrioids, and clear cell and mucinous ovarian cancers, but not in the most common invasive serous cancers [7].

Ovarian cancer can be divided into three main subgroups: epithelial (the most common), germ cell, and sex cord–stromal [8]. There are several morphological categories of epithelial ovarian cancer classified by the WHO (World Health Organization): serous carcinomas (SCs), mucinous carcinomas (MCs), endometrioid carcinomas (ECs), clear-cell carcinomas (CCCs), transitional-cell Brenner tumors, mixed, and undifferentiated type. The most common type of epithelial ovarian cancer is high-grade serous ovarian cancer (HSOC), which constitutes about 85–90% of serous OC cases. Due to an asymptomatic course, vague symptoms of early stages, low effectiveness of diagnostic methods, and a lack of screening tests, most patients are diagnosed at an advanced stage [9]. Moreover, HSOC has a predilection for an unfavorable course and recurrence. Approximately 70% of patients with ovarian cancer develop cancer recurrence or treatment resistance at some stage of cancer development [10].

The ability of cancer to progress and metastasize is caused by phenomena in both tumor cells and the tumor microenvironment. The cancer cells recruit immune cells, vessels, and adipocytes in the tumor environment and reprogram them to promote tumor growth [11]. Moreover, it is also suspected that adipose tissue surrounding the tumor induces chemotherapy resistance and promotes tumor development. Cancers developing in the surrounding adipose tissue, e.g., breast cancer and ovarian cancer, use the energy stored in adipocytes and induce energy metabolism reprogramming in adipocytes [11,12]. The dysregulation of adipose tissue metabolism leads to enhanced expression of hormones, adipokines, inflammatory cytokines, growth factors, enzymes, and free fatty acids (FFAs), which are conducive to cell malignant transformation, proliferation, and angiogenesis [13]. Therefore, it is suspected that adipokines are crucial to the interaction between adipocytes and cancer cells.

Adipokines exert a pleiotropic role in adipose tissue by paracrine signaling but also impact other tissues by endocrine signaling. The most relevant function of adipokines is the regulation of glucose metabolism in the body by modulating insulin sensitivity. Therefore, adipokines play either an anti-hyperglycemic role, e.g., visfatin, irisin, omentin, and adiponectin, or a pro-hyperglycemic role, e.g., leptin, resistin, and chemerin. Moreover, adipokines play a relevant role in inflammation, and some of them exert pro-inflammatory effects, e.g., leptin or resistin, whereas others have anti-inflammatory properties, e.g., adiponectin, omentin, visfatin, and irisin. Interestingly, chronic inflammation in adipose tissue is a vital factor that triggers carcinogenesis.

Previous scientific research has proved increased expression of circulating adipokines, including leptin and apelin, in patients with obesity-related cancers such as breast cancer and prostate cancer. Adipokines play an important role in carcinogenesis by inducing inflammation in the tumor microenvironment [14]. Moreover, adipokines regulate metabolic processes occurring in cancer cells and thus enhance tumor growth [15]. Some adipokines also induce cancer progression by influencing angiogenesis, i.e., the formation of new blood vessels [16]. Many studies focusing on adipokines and the etiopathogenesis of ovarian cancer have shown that adipokines and their receptors are present in both healthy ovaries and ovarian cancer tissues [17]. Adipokines may also have anti-cancer effects. There is a core need to search for pro-cancer adipokines in the realm of cancer prevention and early detection.

Therefore, considering the scarcity of studies showing the relationship between obesity leading to altered adipokines expression and ovarian cancer risk, we would like to define the influence of adipokines on ovarian carcinogenesis. In this review, we seek to outline the indisputable evidence that the role of adipokines is relevant in the reciprocal interaction between cancer and adipocytes. Additionally, we endeavor to define the precise function of several novel adipokines, including chemerin, apelin, visfatin, adiponectin, and resistin in ovarian cancer, and prove whether their action is linked to their direct effect on cancer tissues or is the result of metabolism dysregulation in obesity-related adipose tissue.

## 2. Obesity and Ovarian Cancer Risk

Obesity is associated with excessive calorie intake, resulting in excessive lipid accumulation in adipose tissue. Experimental and clinical studies demonstrated a significant correlation between obesity and the risk of cancer. Numerous meta-analyses pertinent to ovarian cancer epidemiology have shown that obesity can be regarded as a dismal prognostic factor in ovarian cancer. Meta-analyses have demonstrated a strong relationship between BMI and ovarian cancer incidence in low-grade serous tumors, invasive mucinous tumors, premenopausal ovarian cancer, and epidemiological studies [18,19]. Moreover, obesity diagnosed 5 years before the diagnosis of ovarian cancer or obesity diagnosed at a young age has been implicated in ovarian cancer outcomes [20]. However, the research pertinent to high-grade serous ovarian cancer (the most common OC type) has acknowledged that there is no considerable association between elevated BMI and the incidence of high-grade serous ovarian cancer cases [6]. It is worth mentioning that increased BMI is an inaccurate ratio to elucidate visceral fat content—the main source of lipids for ovarian cancer cells, which support cell growth, metastasis, and the production of adipokines. Thus, it would be necessary to assess whether fat accumulation in the environment surrounding the tumor influences OC development and progression.

## 3. A Crosstalk Between Visceral Adipose Tissue, Adipokines, Inflammation, and DNA Damage in Cancer Development

Visceral adipose tissue plays a multifaced role in carcinogenesis. Not only do adipocytes provide energy supplies to cancer cells, but they also extensively produce adipokines that exert a pleiotropic influence on several physiological processes, e.g., angiogenesis and invasion.

The hypertrophy and hyperplasia of adipocytes lead to impaired lipid storage and multiple implications in adipose tissue metabolism, resulting in increased secretion of adipokines and pro-inflammatory cytokines. In carcinogenesis, adipokines produced by adipose tissue induce the influx of immune cells into the tumor, triggering their activation and subsequently initiating chronic inflammation. The action of immune cells in the cancer milieu influences the regulation of tumor development. Leptin, released by adipocytes, diminishes the anti-tumor cytotoxicity of NK cells and, therefore, induces OC development and progression. Moreover, leptin reduces perforin and interferon γ (IFN-γ) secretion by NK cells. Leptin also induces lymphocyte Treg differentiation and increases secretion of pro-inflammatory interleukin 6 (IL-6) and tumor necrosis factor α (TNF-α) by monocytes, which is crucial for cancer progression [21]. Moreover, fat surrounding tumors demonstrates impaired beta-oxidation and increased oxidative stress, leading to enhanced production of reactive oxygen species (ROS) and the damage of DNA structure in cancer cells.

The main genetic changes in ovarian cancer are caused by impairment in the homologous recombination (HR) DNA repair system. Experimental studies have shown that changes induced by obesity in the tumor microenvironment promote DNA damage in breast epithelial cells in carriers of germline *BRCA1* and *BRCA2* mutations. Leptin has also been shown to increase DNA damage in *BRCA* heterozygous human epithelial cells [22]. It seems that other adipokines produced by inflamed adipose tissue may also implicate the DNA repair system. Furthermore, an imbalance between the expression of pro- and anti-inflammatory adipokines leads to the activation of several oncogenic signaling pathways, which may also disturb the DNA repair process [23].

## 4. Adipose Tissue as a Key Factor in Promoting Ovarian Cancer Metastatic Lesions in Peritoneal Cavity

An abundant presence of adipokines was detected in the areas between adipocytes and cancer cells in ovarian metastatic lesions from the peritoneal cavity. It may indicate a predominant role of increased lipid uptake in the reciprocal interaction between cancer and adipocytes [24]. Cancer cells use lipid droplets and long-chain fatty acids stored in adipocytes as a source of energy. The oxidation of fatty acids fulfills the high energy demands, which are vital for rapid tumor growth, mobilization of cancer cells, and metastasis [25].

It was established that adipose tissue of obese or diabetic individuals exerts a more potent effect on cancer development [11]. Recent studies place adipose-derived stem cells (ADSCs) in the limelight of OC peritoneal dissemination. Cancer cells transform ADSCs into cancer-associated adipocytes (CAAs), which present activity comparable to adipocytes of obese or diabetic patients [26]. The CAAs produce mediators such as adipokines and interleukins, which trigger reprogramming and inflammation in the tumor microenvironment. Moreover, CAAs are characterized by increased lipolysis due to an enhanced activity of protein kinase A (PKA). Subsequently, accelerated lipolysis leads to enhanced FA release and influx into cancer cells mediated by cluster of differentiation 36 (CD36). The enhanced CD36 expression in metastatic tissues in omentum highlights the crucial interaction between CD36, adipocytes, and cancer cells, leading to metastasizing and worsening the prognosis [23]. Moreover, it was proved that cancers with a higher abundance of CD36 exhibit unfavorable course [27]. Additionally, CAAs induce AMP-activated protein kinase (AMPK) phosphorylation in cancer cells, leading to increased lipolysis and enhanced expression of enzymes involved in beta-oxidation, i.e., carnitine palmitoyltransferase 1 (CPT1a) and acyl-CoA oxidase 1. The acceleration of beta-oxidation is the source of energy necessary for carcinogenesis.

**Figure 2:**
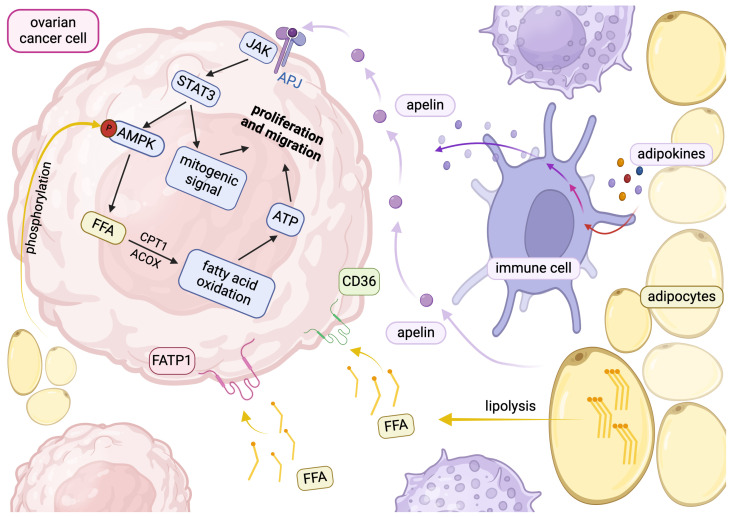
The crosstalk between adipocytes, ovarian cancer cells, and immune cells. Created in BioRender. Kołakowski, A. (2025). https://BioRender.com/j44m979 (accessed on 9 July 2024). Abbreviations: AMPK—adenosine monophosphate-activated protein kinase, ATP—adenosine triphosphate, APJ—apelin receptor, CPT1—carnitine palmitoyltransferase 1, ACOX—acyl-coenzyme A oxidase, FFA—free fatty acid, FATP1—long-chain fatty acid transport protein 1, JAK—Janus-activated kinases, STAT3—signal transducer and activator of transcription 3.

Regarding OC, CAAs induce the activation of the signal transducer and activator of transcription 3 (STAT3) signaling pathway in ovarian cancer cells and thereby initiate autophagy and trigger the expression of genes involved in cell proliferation [28]. This interaction between adipocytes and tumor cells is regulated by the secretion of adipokines. Apelin accelerated cell invasion and mobilization of ovarian cancer cells in in vitro studies. The increased expression of apelin triggered enhanced CD36 expression involved in lipid influx into cells. Therefore, the alteration in lipid metabolism induced by apelin is a hallmark of carcinogenesis. This recognition underlines the crucial role of adipokines in the interaction between cancer and adipocytes, which is mentioned in Figure 2.

## 5. Chemerin

Chemerin, also known as retinoic acid receptor responder 2 (RARRES2), is one of the adipokines suspected of exerting an influence on ovarian cancer. Chemerin is an endogenous leukocyte chemoattractant that induces immunocyte recruitment mediated by its receptors—chemokine-like receptor 1 (CMKLR1), G protein-coupled receptor 1 (GPR1), and CCL chemokine receptor-like 2 (CCRL2). The activation of these receptors induces the influx of specific immunologic cells, especially myeloid, plasmacytoid, and immature dendritic cells (DCs), M1 macrophages, and natural killer (NK) cells producing pro-inflammatory or anti-inflammatory cytokines in the cancer milieu [29]. Cytokines can both upregulate ovarian cancer cell proliferation pathways or inhibit them. The imbalance between the expression of pro- and anti-inflammatory cytokines activates oncogenic pathways, including NF-kB, leading to the malignant transformation of ovarian cells.

The abundant presence of chemerin and its receptors—CMKLR1, GPR1, and CCRL2—has been found in healthy tissues and immune cells but also in many types of cancer, e.g., clear cell renal cell carcinoma, breast cancer, non-small lung cancer, gastric cancer, hepatocellular carcinoma, and colorectal cancer [30,31]. It was acknowledged that chemerin in the cancer microenvironment mediates the influx of circulating plasmacytoid DCs to visceral adipose tissue, where they induce inflammation [32]. It is also a chemoattractant for NK cells, which potentially exert anti-tumor activity. Moreover, chemerin is responsible for the recruitment of macrophages to the tumor site and influences their polarization, affecting tumor progression [33]. The levels of chemerin and its receptors are dependent on the type of cancer and patients’ general state of health. There was a significant increase in chemerin expression in renal dysfunction, diabetes mellitus type 2, obesity, and hypertension. Moreover, the elevated level of chemerin increases the rate of major adverse cardiovascular events [34]. On the contrary, the expression of chemerin significantly declined in adrenocortical carcinoma [35].

The effects of chemerin action in the development of ovarian cancer are contradictory—both anti-cancer and tumor-promoting roles have been demonstrated [36]. In a healthy ovary, chemerin induces apoptosis of ovarian granulosa cells by binding to CMKLR1, suggesting that this phenomenon may also be present in ovarian cancer [37]. The authors of the obesity mouse model study observed that an increase in the chemerin/CMKLR1 axis was associated with oxidative stress and apoptosis biomarkers production. Moreover, increased chemerin levels were observed in the ascitic fluids of patients with ovarian cancer. Additionally, DNA microarray analysis demonstrated that increased chemerin expression was negatively correlated with overall survival in ovarian cancer [29]. The relationship between shorter lifespan and enhanced chemerin expression in ovarian cancer patients suggests that chemerin has pro-cancer properties. Secondly, chemerin is suspected of promoting metastases. Metastasizing in OC differs from metastasizing via the blood-derived route because it is widely correlated with the dysfunction of cell connectivity, cell motility, and dissemination through the peritoneal fluid. A body of evidence suggests that chemerin exerts a pro-cancer effect in OC via increasing programmed death ligand-1 (PD-L1) expression and enhances the proliferation and migration of OC cells. There was a considerable increase in the presence of chemerin and PD-L1 in ovarian cancer. Chemerin increases PD-L1 expression by upregulation of p38 and extracellular signal-regulated kinase 1/2 (ERK1/2) phosphorylation, leading to enhanced IL-6 expression and concomitantly enhancing the expression of PD-L1. Induced PD-L1 expression increases the proliferation and motility of ovarian cancer cells and, therefore, promotes peritoneal dissemination and metastasis. It was also shown that the metastatic ovarian cancer cell line exhibited an enhanced chemerin expression compared to the normal ovarian carcinoma cell line. Moreover, the chemerin expression was significantly increased in ascites compared to the serum in ovarian cancer patients. This recognition indicates a local increase in chemerin expression in the surroundings of OC [38]. This indicates that the expression of chemerin is important in ovarian cancer and metastasis formation. Moreover, studies conducted on ovarian cancer cell lines have demonstrated that chemerin initiates the CMKLR1/Ras homolog family member A (RhoA)/rho-associated, coiled-coil-containing protein kinase 1 (ROCK) cascade, which contributes to the migration and epithelial–mesenchymal transition (EMT) of ovarian cancer cells [39].

On the other hand, in studies conducted on other tumors, including prostate cancer and sarcoma, the tumor lines have shown that chemerin induces an opposite effect on the expression of PD-L1. Chemerin influenced the phosphatase and tensin homolog deleted on chromosome ten (PTEN) pathway and, therefore, suppressed cancer cell migration and diminished cancer development in vivo [40]. The in vitro studies conducted on ovarian cancer cells have also demonstrated the anti-cancer effect of chemerin. More precisely, there was a significant decrease in the number of ovarian cancer cells after the incubation of the ovarian cancer cell line with chemerin. A recent study demonstrated that chemerin induced a noticeable upregulation of interferon α-related gene (INFα-related gene) expression in ovarian cancer, which suppressed ovarian cancer growth [41]. Moreover, it was acknowledged that an augmented level of CMKLR1 in ovarian cancer specimens improved the outcome in individuals with ovarian cancer [42]. Additionally, Hoffmann et al. revealed that chemerin administration did not exert influence on both ovarian cancer cells and physiological ovarian cells [43].

Chemerin plays a pivotal role in the development of inflammation in cancer. There is a lack of studies assessing the relationship between chemerin expression and the production of cytokines in ovarian cancer. A vast number of studies have linked increased expression of CCRL2—one of the chemerin receptors—and increased levels of pro-inflammatory cytokines such as interleukin 1β (IL-1β), TNF-α, IL-6, and IFN-γ. This is consistent with the fact that chemerin induces the secretion of pro-inflammatory cytokines in other cancers. Oral squamous cell carcinoma (OSCC) samples with cervical lymph node metastasis exhibit elevated levels of chemerin, IL-6, interleukin 15 (IL-15), granulocyte macrophage-colony stimulating factor (GM-CSF), regulated upon activation, normal T cell expressed and secreted (RANTES), TNF-α, and vascular endothelial growth factor (VEGF) compared to samples without lymph node metastasis. Moreover, this research indicates a correlation between chemerin expression and IL-6, GM-CSF, TNF-α, and VEGF expression [14]. On the contrary, the attenuation of chemerin action in rhabdomyosarcoma (RMS) led to increased production of pro-inflammatory cytokines such as IL-1β, IL-6, interleukin 10 (IL-10), and TNF-α [44]. For this reason, we cannot determine the pivotal role of chemerin in inflammation development in the cancer milieu.

There are also reports that chemerin influences cancer development by inducing angiogenesis. A hallmark feature of chemerin in angiogenesis is its ability to induce VEGF expression. Moreover, recent research revealed that chemerin triggers neovascularization similarly to VEGF [16]. Unfortunately, the role of chemerin in angiogenesis in patients with ovarian cancer has not yet been investigated. Moreover, the available information on the action of chemerin in ovarian cancer is limited and ambiguous. Thus, it should be emphasized that the topic of serum chemerin levels and the expression of its receptors in ovarian cancer tissue requires further research as a new potential therapeutic target.

## 6. Apelin

Apelin, one of the adipokines, exerts a pleiotropic effect on metastasizing to the peritoneum. Apelin expression was increased in numerous pathological conditions in the human body, including obesity, diabetes, and cancers [45]. Novel research proved that apelin induces the influx of T CD8+ and CD4+ lymphocytes to the central part of the tumor but does not impact other immune cells. Altered immune cell localization may result from the specific properties of colon cancer. In this study, apelin action led to anti-cancer effects [46]. Moreover, another study indicated that apelin inhibits the phagocytic activity of macrophages in the peritoneum [47]. Recent research revealed enhanced expression of apelin, assessed by immunohistochemistry (IHC) methods, in obese ovarian cancer patients compared to non-obese individuals. A significant relationship was found between BMI, overall survival, and apelin expression in this study [48]. Previous scientific research has shown an increased expression of circulating adipokines—leptin and apelin—in obesity-related cancers such as breast cancer and prostate cancer [49,50,51,52]. As mentioned in previous paragraphs, apelin influences lipid metabolism in cancer cells, thereby increasing the ability of cancer cells to migrate and form metastases.

Apelin exerts influence on lipid uptake into cancer cells, which is mediated by the apelin receptor (APJ). The activation of the APJ receptor induces the STAT3 pathway, leading to increased CD36 expression, which causes increased lipid transport and lipid droplet accumulation in ovarian cancer cells. Moreover, apelin accelerates fatty acid oxidation mediated by the activation of the AMPK-CPT1a pathway [24]. Furthermore, induction of CPT1a, the rate-limiting enzyme of fatty acid β-oxidation, increases fatty acid utilization. There was a significant increase in the level of CPT1a in metastases compared to primary colorectal cancer. Moreover, a body of evidence suggests that accelerating fatty acid oxidation provides energy for cancer cells and, therefore, promotes metastases formation [53].

Analyzing research on the role of apelin in ovarian cancer cells, it can be concluded that apelin exerts an impact on cancer cell migration and acts as a mitogenic factor. It was acknowledged that increased expression of the apelin receptor caused cancer cell relocation and migration to the omentum [24]. A body of evidence suggests that there is a significant association between the role of apelin and peroxisome proliferator-activated receptor gamma (PPAR) in ovarian cancer pathogenesis. It was observed that bisphenol A-induced apelin expression in ovarian cancer cell lines activates cell proliferation via peroxisome proliferator-activated receptor gamma [54].

Moreover, the relationship between the expression of apelin and the role of poly-ADP ribose polymerase (PARP) in carcinogenesis has been demonstrated in studies on several cancer types. Apelin suppressed apoptosis of adenocarcinoma cell lines by attenuation of PARP protein degradation [55]. Moreover, the current state of knowledge acknowledges that PARP is a nuclear receptor involved in regulating cell division, translational and post-translational modification of proteins, and DNA repair system [56]. The abundant presence of PARP was revealed in ovarian carcinomas and benign ovarian tumors, but also in healthy ovaries. PARP receptor inhibitors (olaparib, niraparib, and rucaparib) are used as a maintenance treatment for platinum-sensitive relapsed OC but also in homologous recombination deficiency (HRD) patients [57]. However, recent studies proved that PARP inhibition suppresses solute carrier family 7 member 11 (SLC7A11), resulting in decreased glutathione production. The downregulation of glutathione production leads to lipid peroxidation and the iron-dependent death of ovarian cancer cells, called ferroptosis. This mechanism leads to resistance to olaparib—one of the PARP inhibitors—and shows that ferroptosis inhibition should be implemented with PARP inhibitors in ovarian cancer treatment [58]. Recent reports on the effect of apelin on PARP expression also show that apelin is an interesting target for research on the effectiveness of PARP inhibition in the treatment of ovarian cancer.

However, recent studies have also acknowledged that apelin exerts an anti-cancer effect on ovarian cancer cell lines. However, the exact mechanism induced by apelin is not related to the direct activation of proteins involved in apoptosis of epithelial (OVCAR-3) and granulosa (COV434) ovarian cancer cell lines. Apelin suppresses the proliferative effect of estrogen on epithelial (OVCAR-3) ovarian cancer cell lines and insulin-like growth factor 1 (IGF-1) on granulocyte (COV434) ovarian cancer cell lines by the interaction of the apelin receptor with those hormones’ receptors [59].

Other studies yield inconsistent results regarding the role of apelin in ovarian cancer because the expression of the apelin was negatively correlated with the expression of its receptor. Moreover, increased expression of apelin receptors and decreased expression of apelin was demonstrated in epithelial ovarian cancer compared to granulosa tumors [54]. Recent research has also revealed the enhanced expression of apelin and moderate expression of APJ assessed by immunohistochemistry in patients with advanced-stage serous carcinoma, both with and without lymphatic metastases. A significant relationship was found between BMI, overall survival, and apelin expression in this research [48].

## 7. Visfatin

Visfatin is one of the adipokines involved in ovarian cancer pathogenesis. Scientific research conducted so far has shown that visfatin can act as nicotinamide phosphoribosyltransferase (NAMPT) and is its extracellular form [60]. Enhanced expression of visfatin was detected in visceral adipose tissue in obese patients, whereas decreased visfatin expression was found in subcutaneous adipose tissue. The Cancer Genome Atlas (TCGA) proved that elevated NAMPT expression has a significant relationship with dismal prognosis in ovarian cancer patients [61].

Regarding the role of visfatin in ovarian tumors, it was suspected of reprogramming glucose metabolism in ovarian granulosa tumors and promoting invasiveness of ovarian cells. A body of evidence suggests that visfatin shifts glucose metabolism into anaerobic processes, which is a hallmark for cancer cells [62]. Firstly, visfatin enhanced anaerobic utilization of glucose, upregulation of glucose transporter 1 (GLUT1), and glucose uptake by granulosa cell tumor-derived spheroid cells and elevated the expression of glycolytic enzymes in adult granulosa cell tumor-derived spheroid cells. The level of visfatin was elevated in ascites compared to serum in ovarian cancer patients. Previous studies have revealed that visfatin induces matrix metallopeptidase 2 (MMP2) expression in ovarian granulosa cell tumors, which involves extracellular matrix degradation (a crucial step for metastatic progression) and suppresses claudin 3 and 4 expression, which is a component of tight junction strands [62].

The role of visfatin and its intracellular form—NAMPT—is also associated with PARP, similar to apelin. Besides regulating apoptosis, PARP is also involved in DNA repair processes. NAMPT is a crucial enzyme in the cells that converts nicotinamide to nicotinamide mononucleotide (NMN), which is then a substrate for nicotinamide adenine dinucleotide (NAD+) synthesis. NAD+ is a vital substrate for protein modifications, including poly-ADP-ribosylations catalyzed by PARP and deacetylation of chromatin by sirtuins. All these reactions are crucial for genome stability and DNA repair. Recent studies have acknowledged that regulation of NAMPT production influences PARP action [63]. NAD+ facilitates DNA double-strand breaks repair via PARP-dependent processes [64]. Moreover, in high-grade serous ovarian cancer, NAD+ suppression prevents the development of resistance to PARP inhibitors. The combination of a PARP inhibitor and an NAMPT inhibitor initiated DNA degradation and caspase-3 cleavage, leading to apoptosis of ovarian cancer cells. All these phenomena diminished ovarian cancer growth [65]. However, in the studies conducted on ovarian cancer cell lines, it was shown that visfatin did not exert an impact on PARP1 expression [66].

Moreover, it was also revealed that visfatin exerts an influence on ovarian cancer cell susceptibility to chemotherapy. It was proved that chemotherapy based on cisplatin triggers the activation of cancer stem-like cells, which contributes to chemoresistance. Interestingly, NAMPT suppression ameliorates the adverse effects induced by cancer stem-like cells and hinders OC cell development treated with cisplatin chemotherapy in vitro and in vivo. Moreover, combined therapy based on an NAMPT inhibitor—FK866—and cisplatin enhanced the OC overall survival in animal models. Moreover, NAMPT inhibition decreased enhanced aldehyde dehydrogenase action triggered by cisplatin [61].

Visfatin also triggered ovarian cancer resistance to anoikis, which is a specific type of apoptosis typical for circulating cancer cells that lose connectivity with the intercellular matrix and participate in the spread of the tumor and the formation of metastases. The suppression of anoikis of ovarian cancer spheroids is regulated by visfatin via modulating mitochondrial activity. It was acknowledged that visfatin exerts an anti-apoptotic effect on ovarian cancer cells by suppressing caspase-3 expression. Moreover, visfatin accelerated mitochondrial activity, and FK866 (visfatin antagonists) hindered mitochondrial activity [66].

Visfatin also exerts an effect on ovarian cancer invasiveness. It was established that visfatin expression was increased in ovarian cancer patients, and visfatin expression in ascites was enhanced compared to expression in serum in ovarian cancer individuals. Moreover, there was also a significant relationship between the level of visfatin in ovarian cancer patients and the presence of metastases in the omentum. Visfatin, derived from ascites, impacted ovarian cancer cells by enhancing migration through the polymerization of actin filaments, resulting in the formation of lamellipodia and filopodia. The alterations in actin configuration were initiated by the activated Rho/ROCK pathway. The suppression of actin polymerization hindered the development of cancer cell motility. Therefore, a body of evidence suggests that visfatin plays a pivotal role in migration and metastasizing to peritoneum [67].

## 8. Resistin

The abundant presence of resistin is widely correlated with pathological conditions in the human body, e.g., obesity, insulin resistance, diabetes, and polycystic ovary syndrome [68,69,70]. The recent meta-analyses acknowledged that there is a remarkable correlation between the enhanced expression of resistin and the incidence of cancers associated with obesity [70]. Resistin exerts influence on different immune cells. Resistin is involved in the regulation of immune cell function, especially macrophages and T cells. Resistin declines the reactions of DCs with antigens and the cytokine release by dendritic cells. It also enhances the role of regulatory T cells [33].

The novel scientific reports also demonstrate that the resistin expression is enhanced in ovarian cancer tissues. Regarding the role of resistin on ovarian cancer cells, it enhances the proliferation of ovarian cancer cells by triggering the mammalian target of rapamycin kinase (mTOR) pathway and upregulating 70 kDa S6 kinase responsible for autophagy inhibition [71]. Resistin also accelerates ovarian cancer cell migration by triggering MMP2 expression. Resistin also plays a crucial role in angiogenesis because it was established that ovarian cancer cell lines treated with resistin exhibit an increase in VEGF production [72].

Regarding the clinical significance of resistin on ovarian cancer, the current scientific reports indicate that resistin induces chemoresistance to cisplatin. The novel research demonstrated that overexpression of microRNA (miRNA), e.g., pre-let-7a, pre-miR-200c, and pre-miR-186 attenuated resistin action in ovarian cancer, leading to epithelial-to-mesenchymal transition, which is a hallmark of cancer cell migration [72]. Moreover, there is also compelling evidence that resistin production is remarkably associated with ovarian cancer prognosis. There is a significant relationship between the enhanced resistin expression and the grade and lymph node metastasis of OC [71]. 

**Figure 3:**
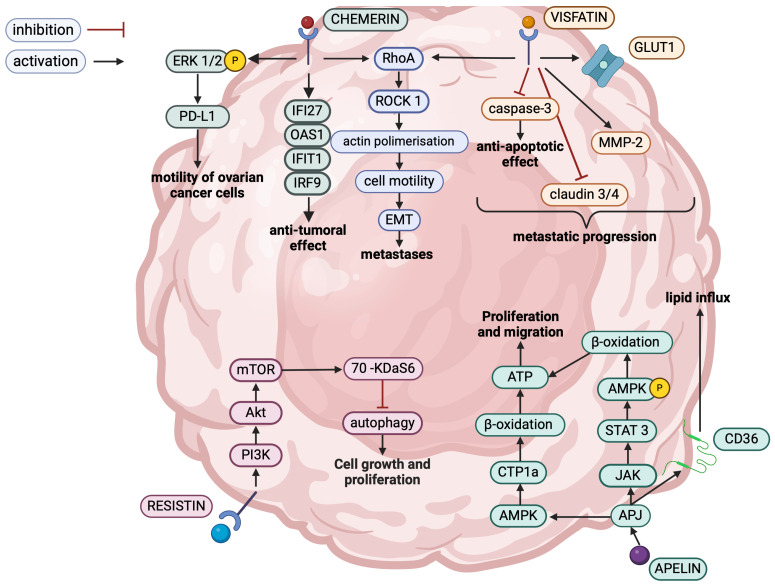
The complex impact of adipokines on the ovarian cancer cell. Created in BioRender. Kołakowski, A. (2025). https://BioRender.com/o89r036 (accessed on 9 July 2024). Abbreviations: RhoA—Ras homolog family member A; ROCK 1—rho-associated, coiled-coil-containing protein kinase 1; EMT—epithelial–mesenchymal transition; *IFI 27*, *OAS1*, *IFIT1*, *IRF9*—interferon alfa response genes; ERK 1/2—extracellular signal-regulated kinase 1/2; PD-L1—programmed cell death ligand 1; MMP-2—matrix metalloproteinases 2; GLUT 1—glucose transporter 1; mTOR—mammalian target of rapamycin; PI3K—phosphoinositide 3-kinases; Akt—serine/threonine kinase 1; 70 KDaS6—70 kDaS6 kinase; AMPK—adenosine monophosphate-activated protein kinase; CTP1a—carnitine palmitoyltransferase 1A; ATP—adenosine triphosphate; JAK—Janus kinase; STAT 3—signal transducer and activator of transcription 3; CD 36—cluster of differentiation 36 or fatty acid translocase; APJ—apelin receptor.

The Figure 3 shows signaling pathways mediated by adipokines in ovarian cancer cells and Table 1 sums up the influence of adipokines on ovarian cancer cells.

## 9. Future Directions

Several miRNAs have been shown to exert a significant impact on ovarian cancer. The mechanism of miRNA action is linked to cancer-associated adipocytes. Dysfunctional adipocytes release the exosomes consisting of diverse miRNAs, lipids, growth factors, and receptors. These miRNAs play a relevant role in the progression of metabolic syndrome and obesity-related cancers. Exosomal miRNA, including miR-23a and miR-23b, released by white adipose tissue influence cancer cell progression. Moreover, exosomal miRNAs are also transported from cancer cells to cancer-related adipocytes and induce lipolysis in adipocytes [73]. Novel research pertinent to noncoding RNAs in ovarian cancer revealed that expression of miRNA-205 was markedly augmented in ovarian cancer patients. Moreover, there was a remarkable increase in the expression of miRNA-205 in circulating exosomes in metastatic ovarian cancer. While investigating the role of miRNA-205, it was proved that miRNA-205 promotes angiogenesis via the PTEN-AKT pathway and the growth of ovarian cancer. Therefore, it induces metastasizing of ovarian cancer cells [74].

There is also evidence that noncoding RNAs and adipokines are engaged in mutual interaction and play significant roles in ovarian cancer. miR-204-5p, derived from breast cancer, induces the production of leptin by cancer-related adipocytes. Leptin induces a signaling pathway in white adipose tissue, leading to lipolysis. Therefore, this phenomenon leads to cachexia and worsens the prognosis of patients [75]. Moreover, visfatin enhanced miR-21 expression and, therefore, induced the motility of hepatocellular carcinoma cells. Moreover, it was acknowledged that miRNA-1908 exerts an influence on the pathogenesis of obesity and cancers [76]. The expression of miR-1908 is regulated by diverse factors, including TNF-α, leptin, and resistin, as well as FFA. TNF-α enhanced the expression of miR-1908 in the human adipocytes, but adipokines (resistin and leptin) and FFAs markedly declined the expression of miR-1908 [77]. Recent research has revealed that the expression of miR-1908 is decreased in the serum of high-grade serous ovarian cancer patients [78]. However, the precise mechanism leading to the downregulation of miR-1908 in high-grade serous ovarian cancer is not well investigated. Novel studies proved that the declined expression of miR-1908 was correlated with poor prognosis in ovarian cancer [79]. In other cancers, including glioma and osteosarcoma, miR-1908 enhances the level of PTEN and stimulates the PI3K/Akt signaling pathway [80,81]. These processes accelerate cancer cell proliferation and migration and induce angiogenesis. Therefore, the roles of miRNAs and their associations with adipokines should also be investigated in ovarian cancer. Moreover, serum miRNAs, especially those associated with adipokines, constitute a potential target for ovarian cancer diagnosis [82]. Therefore, the role of miRNAs as a diagnostic tool should be investigated in ovarian cancer.

There is a vital need to improve the effectiveness of ovarian cancer treatment by considering novel mechanisms of ovarian cancer progression. The current scientific reports demonstrated that adipokines induce ovarian cancer progression. However, there is more potent evidence of a direct effect of adipokines on the ovaries in experimental studies. Therefore, more detailed research on this effect should be carried out in clinical trials. There is a need to conduct further research to investigate adipokine inhibitors and elucidate the effects of adipokine inhibition on ovarian cancer.

There is compelling evidence that the use of inhibitors of adipokines or their pathways resulted in the attenuation of ovarian cancer proliferation and the inhibition of cancer cell relocation and migration to the omentum, as demonstrated in the following recent publications: ‘Adipokine Apelin/APJ Pathway Promotes Peritoneal Dissemination of Ovarian Cancer Cells by Regulating Lipid Metabolism’, ‘Chemerin promotes proliferation and migration of ovarian cancer cells by upregulating expression of PD-L1’, ‘Inhibition of nicotinamide dinucleotide salvage pathway counters acquired and intrinsic poly(ADP-ribose) polymerase inhibitor resistance in high-grade serous ovarian cancer’, and ‘Resistin Expression in Epithelial Ovarian Cancer promotes the Proliferation and Migration of Ovarian Cancer Cells to Worsen Prognosis’.

In research conducted by Dogra et al., it was demonstrated that apelin antagonists inhibited ovarian cancer cell relocation and migration to the omentum [24]. Furthermore, the combination of a PARP inhibitor and an NAMPT inhibitor initiated DNA degradation and caspase-3 cleavage, leading to apoptosis of ovarian cancer cells [65]. Small interfering RNA (siRNA) implemented in cancer cells to inhibit PD-L1 expression significantly suppressed chemerin-induced ovarian cancer cell proliferation [38]. Moreover, rapamycin, a well-established inhibitor of mTOR/p70S6K, inhibits resistin-induced ovarian cancer cell proliferation by blocking 70 kDa S6 kinase, which is a key element of mTOR signaling [71].

Therefore, there is a need to conduct clinical trials with adipokine antagonists to elucidate their effectiveness as additional therapy in ovarian cancer patients.

## 10. Conclusions

The current state of knowledge indicates that the pathogenesis of ovarian cancer is widely dependent on interaction with the adipose tissue environment. Adipose stem cells (ADSCs) play a pivotal role in OC pathogenesis and omental metastasizing. The alterations in lipid deposition in ovarian cancer individuals trigger the secretion of diverse adipokines from both adipocytes and immune cells. Novel research pertinent to ovarian cancer revealed that some adipokines exhibit both pro-cancer and anti-cancer effects. However, there is more potent scientific evidence that adipokines promote ovarian cancer progression. Moreover, some adipokines are gaining gradual recognition as unfavorable prognostic factors in ovarian cancer. Adipokines influence metabolic reprogramming, migration, and inflammation initiation in ovarian cancer. Moreover, adipokines affect the DNA repair system, leading to genomic instability, which is a hallmark of carcinogenesis. Furthermore, the enhanced activity of ascites-derived adipokines is one of the factors that contribute to peritoneal dissemination in ovarian cancer.

Novel research indicates that the suppression of adipokine action by their antagonists hindered apoptosis of ovarian cancer cells and diminished ovarian cancer growth. Moreover, the suppression of adipokines enhanced the effect of chemotherapy in recent trials. The improvement in overall survival in ovarian cancer individuals treated with cisplatin after implementing visfatin antagonists to the therapy indicates that adipokines pose a potential therapeutic target for individual ovarian cancer treatment strategies.

## Figures and Tables

**Table 1:** The effect of adipokines on ovarian cancer development.

Type of Adipokine	The Effect on Ovarian Cancer	Mechanism	Reference
Chemerin	Pro-cancer effect	Increase in programmed cell death ligand 1 (PD-L1) expression and enhances proliferation and migration of OC cells	[38]
Chemerin	Suppression of ovarian cancer growth	Upregulation of interferon α-related gene (INFα-related gene) expression in ovarian cancer	[41]
Apelin	Anti-cancer effect	Apelin suppresses the proliferative effect of estrogen on epithelial (OVCAR-3) ovarian cancer cell lines and insulin-like growth factor 1 (IGF-1) on granulocyte (COV434) ovarian cancer cell lines by the interaction of the apelin receptor with those hormones’ receptors	[59]
Apelin	Mitogenic factor	Bisphenol A-induced apelin expression in ovarian cancer cell lines activates cell proliferation via peroxisome proliferator-activated receptor gamma	[54]
Visfatin	Anti-apoptotic effect on ovarian cancer cells	Suppression of caspase-3 expression	[66]
Visfatin	Pro-cancer effect	Enhanced anaerobic utilization of glucose, upregulation of glucose transporter 1 (GLUT1) and glucose uptake by granulosa cell tumor-derived spheroid cells, and elevated the expression of glycolytic enzymes in adult granulosa cell tumor-derived spheroid cells	[62]
Visfatin	Pro-metastatic effect	Induction of matrix metallopeptidase 2 (MMP2) expression in ovarian granulosa cells and suppression of claudin 3 and 4 expression	[62]
Visfatin	Pro-metastatic effect	Activation of the Ras homolog family member A (RhoA)/rho-associated, coiled-coil-containing protein kinase 1 (ROCK) pathway, leading to the polymerization of actin filaments, thereby resulting in lamellipodia and filopodia formation and the development of cancer cell motility	[67]
Resistin	Pro-cancer effect	Enhanced proliferation of ovarian cancer cells through the induction of the mammalian target of rapamycin kinase (mTOR) pathway and the upregulation of 70 kDa S6 kinase	[71]
